# Double trouble: renal dioctophymosis and chronic diaphragmatic hernia in an asymptomatic dog treated laparoscopically

**DOI:** 10.1007/s11259-026-11103-9

**Published:** 2026-02-28

**Authors:** Pâmela Caye, Jean Carlos Gasparotto, Brenda Viviane Götz Socolhoski, Amanda Oliveira Paraguassú, Otávio Henrique de Melo Schiefler, Gabryelle Ferreira da Silva, Cinthia Melazzo de Andrade, Maurício Veloso Brun

**Affiliations:** 1 Programa de Pós-Graduação em Medicina Veterinária, Universidade Federal de Santa Maria, Santa Maria, Rio Grande do Sul Brazil; 2 Faculdade de Veterinária, Universidade Federal do Rio Grande do Sul, Porto Alegre, Rio Grande do Sul Brazil; 3 Curso de Medicina Veterinária, Centro de Ciências Rurais, Universidade Federal de Santa Maria, Santa Maria, Brazil; 4 Departamento de Clínica de Pequenos Animais, Universidade Federal de Santa Maria, Santa Maria, Brazil

**Keywords:** *Dioctophyme renale*, Nephrectomy, Videolaparoscopy, Giant kidney worm, Diaphragmatic defect

## Abstract

Dioctophymosis, caused by the nematode *Dioctophyme renale*, is a parasitic disease that leads to progressive destruction of the renal parenchyma and is most effectively treated with surgical nephrectomy. Chronic diaphragmatic hernias are characterized by defects in the diaphragm, most commonly associated with traumatic events, and require surgical correction. Although both conditions can be managed using minimally invasive techniques, laparoscopic approaches remain uncommon in veterinary practice, and there are no previous reports of combined surgical treatment of these two conditions in a single procedure. This report describes the first case of simultaneous laparoscopic management of renal dioctophymosis and chronic diaphragmatic hernia in a 10-year-old asymptomatic dog. The patient was diagnosed with *D. renale* infestation in the right kidney through abdominal ultrasonography and underwent a three-port laparoscopic nephrectomy. During the procedure, a diaphragmatic defect with herniation of perirenal fat was incidentally identified and repaired using a barbed suture. The surgery was completed successfully without the need for conversion to an open approach. Postoperative recovery was favorable, despite transient hypoglycemia-associated seizures, and the dog was discharged four days after surgery. Laparoscopy proved to be a safe, minimally invasive, and effective option for the simultaneous treatment of renal dioctophymosis and chronic diaphragmatic hernia in dogs.

## Introduction

Dioctophymosis is a parasitic disease with worldwide occurrence and zoonotic characteristics. It is caused by the nematode *Dioctophyme renale*, a worm that responds poorly to conventional antiparasitic treatments (Pedrassani and Nascimento 2015; Perera et al. 2021). It reaches considerable size and shows a predilection for the kidneys, often leading to organ loss. Therefore, definitive treatment is typically surgical (Sapin et al. 2017; Caye et al. 2024a, b). Affected patients are usually asymptomatic, making dioctophymosis a serious and often silent disease (Amaral et al. 2020; Caye et al. 2024b).

Chronic diaphragmatic hernias (CDH) are diaphragmatic defects, usually of traumatic origin, that were not immediately corrected. Like dioctophymosis, they require surgical treatment and can be asymptomatic (Minihan et al. 2004). Both diseases can be treated with open or laparoscopic surgery (Minihan et al. 2004; Caye et al. 2023, 2024b), but there are no reports associating both treatments in a single surgical procedure in dogs. This work describes a laparoscopic nephrectomy in a dog with dioctophymosis in the right kidney, where there were intraoperative diagnosis and correction of a chronic diaphragmatic hernia.

## Case report

The extreme floods of May 2024 in southern Rio Grande do Sul, Brazil, affected thousands of people (Marengo et al. 2024), as well as thousands of dogs, particularly those in socially vulnerable areas. These same areas have a high number of dioctophymosis diagnoses in dogs (Rappeti et al. 2016). In this context, a dog with an estimated age of 10 years, from a fishing community, was diagnosed with *D. renale* in the right kidney. The patient was neutered but had no prior medical history available, as it had not been clinically evaluated since its adoption by the owners.

The animal exhibited normal behavior, weighed 28 kg, had mild locomotion difficulty, and intermittent hematuria. There were no other clinical signs or history of trauma. Abdominal ultrasonography indicated the presence of *D. renale* in the right kidney, and hematological evaluations showed nothing noteworthy. Urinalysis revealed hematuria, leukocyturia, and parasite eggs as the most relevant alterations.

The patient was referred for laparoscopic nephrectomy with three ports in the right flank, with 8 mmHg pneumoperitoneum, as described by Caye et al. (2023). The kidney appeared large and flaccid, which hindered exposure of the renal hilum. The renal vein was dissected, but isolation of the renal artery proved challenging. In the authors’ experience, it is preferable to occlude arterial blood flow before the vein, as it prevents renal engorgement. However, this sequence was not possible, so the vein was sectioned first, followed by a structure consistent with a nonpulsatile renal artery, using an ultrasonic scalpel.

However, during subsequent manipulation, the kidney developed a diffuse subcapsular hematoma with increased volume, raising suspicion that the renal artery had not been previously transected or that an accessory renal artery was present. The kidney was retracted medially, allowing inspection of its right lateral surface and adhesiolysis. At this point, perirenal fat adherent to the diaphragmatic insertion with the right muscular wall was visualized abnormally. Upon manipulation, the fat was easily reduced and removed from inside the thorax through an approximately 1 cm opening. After fat removal, the diaphragm became flaccid due to pneumothorax. The abdominal insufflation pressure was reduced to 5 mmHg.

**Fig. 1:**
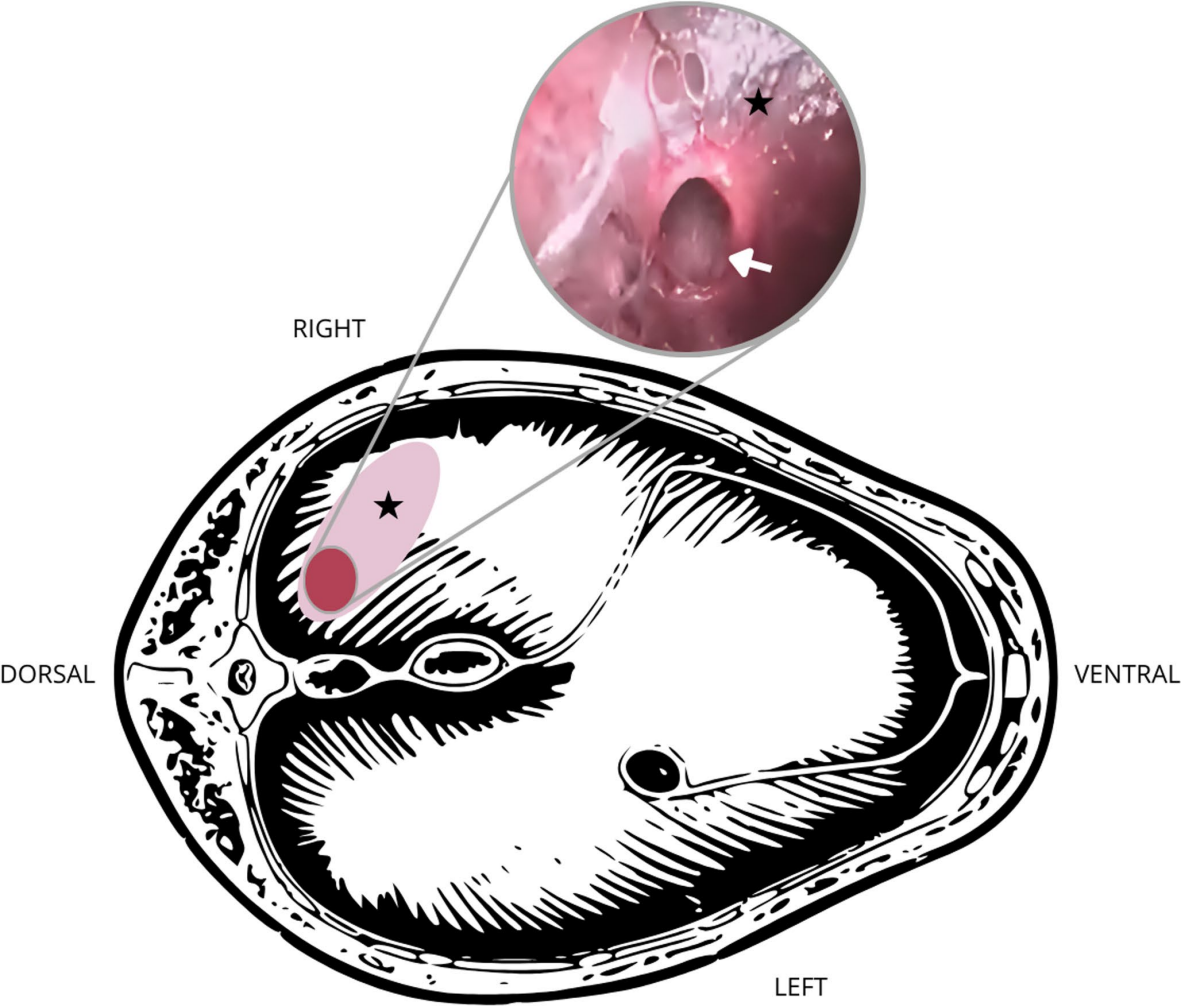
Schematic representation of the caudocranial view of the diaphragm of a dog positioned in left lateral recumbency, with right renal dioctophymosis and a chronic diaphragmatic hernia. The pink area indicates the site of diaphragmatic rupture covered by peritoneum (asterisk), and the red area represents the region where perirenal fat was herniated into the thoracic cavity (highlighted image). The white arrow indicates the interior of the thoracic cavity

The diaphragm was evaluated and showed extensive rupture of muscle bundles, though covered by peritoneum (Fig. 1). This rupture was located on the right side of the diaphragm, near the muscular insertion. The only opening was near the kidney and had been occluded by the perirenal fat. A 10 mm, 30-degree laparoscope was advanced through the lesion, and the thorax was inspected, showing no signs of effusion, pulmonary atelectasis, adhesions, abdominal organs, or parasites. After inspection, the diaphragmatic lesion was enlarged to expose the muscle bundles for correction.

Using a needle holder and a counter needle holder, the wound was closed in a simple continuous pattern with a 0 barbed suture (V-Loc™). Before complete hernia closure, a drain was placed in the right flank and inserted into the thorax through the diaphragmatic wound. Negative thoracic pressure was immediately restored. Subsequently, a standard laparoscopic nephrectomy was performed under a pneumoperitoneum maintained at 5 mmHg. Perirenal adhesiolysis, hemostasis of a structure consistent with a renal artery embedded within the perirenal fat, and ureteral transection were carried out using an ultrasonic scalpel. The kidney was placed in a specimen retrieval bag and extracted through the medial incision. The surgical wounds were closed in three layers.

The patient recovered from anesthesia with the thoracic drain still in place, which was removed after three nonproductive drainages within two hours. Postoperatively, the patient received analgesic and anti-inflammatory medications and had a good recovery during the initial postoperative hours. However, approximately 24 and 48 h later, the patient experienced two episodes of epileptic seizures. Hypoglycemia was suspected, as blood glucose was measured at 45 mg/dL despite normal food intake. Following this period, blood glucose levels normalized, and no further seizure activity was observed. Further investigation into the underlying cause of the seizures was not pursued due to limited resources. No additional anticonvulsant therapy was required, and the patient continued to recover uneventfully, being discharged four days after surgery.

## Discussion and conclusions

Asymptomatic diseases pose a significant clinical challenge for veterinarians. This challenge is amplified when patients show no specific clinical signs for two concurrent conditions. Patients with *D. renale* often show no signs perceptible to their owners (Amaral et al. 2020). Hematuria, as presented in this case, is one of the most common findings in dogs with dioctophymosis (Milech et al. 2022; Caye et al. 2024a, b). Although nonspecific, the presence of blood in urine in dogs from regions susceptible to renal parasitosis should always be investigated.

Acquired CDH is frequently associated with severe trauma, such as impact from vehicular accidents (Shao et al. 2024). However, without a patient history, the chronicity of the condition may be impossible to determine. When abdominal organs are present in the thorax, imaging exams aid in diagnosis. Simple or contrast radiographs, ultrasounds, and computed tomography (CT) can be performed (Minihan et al. 2004; Shao et al. 2024). Due to the absence of clinical signs and limited financial resources, the treated patient did not undergo thoracic imaging. Nevertheless, considering the small portion of herniated fatty content and absence of pneumothorax, it is likely that a preoperative CDH diagnosis would not have been achieved.

Although unreported in the literature, in the authors’ experience, CDH can also be diagnosed during abdominal surgical procedures. In observed cases, upon abdominal incision, patients exhibited respiratory changes due to the consequent development of pneumothorax. In this case, the patient showed no respiratory or hemodynamic alterations, even with an 8 mmHg pneumoperitoneum, because the hernia was tamponaded by perirenal fat. The preservation of the peritoneum covering the injured muscle also promoted respiratory stability. Thus, pneumothorax and diaphragmatic flaccidity only occurred after fat removal. With pneumoperitoneum reduced to 5 mmHg and careful anesthetic and ventilatory management, the patient experienced no significant intraoperative complications and could undergo laparoscopic herniorrhaphy.

The CDH diagnosis was favored by the coincidence that the parasitized kidney was on the right side, the same location as the CDH. Had the lesion been on the left side of the diaphragm, patient positioning would have posed particular challenges for diagnosis and especially for treatment. As an asymptomatic patient who only developed pneumothorax after manipulation, this condition might have gone unnoticed if the CDH had been located elsewhere.

To avoid alterations from pneumoperitoneum and pneumothorax during laparoscopic herniorrhaphy, *gasless* techniques without abdominal insufflation have been developed. In these procedures, the working space for diaphragmatic suturing is created by abdominal wall traction (Brun et al. 2022). These techniques offer benefits regarding respiratory and hemodynamic stability (Koivusalo et al. 1998). In this case, *gasless* techniques were not employed because the CDH finding was accidental. The authors recommend using *gasless* laparoscopic surgical devices for pre-diagnosed hernias.

Although laparoscopic diaphragmatic herniorrhaphy requires advanced skills, the use of barbed suture facilitated the procedure’s success. The barbed suture reduced surgical time compared to conventional laparoscopic suturing while ensuring secure wound closure. The availability of appropriate materials and training in intracorporeal suturing was crucial for therapeutic success and avoiding conversion to open surgery. Thus, this represents the first reported case of fully laparoscopic, single-stage treatment for renal dioctophymosis and chronic diaphragmatic hernia in a dog.

It can be concluded that laparoscopy for treating dioctophymosis and chronic diaphragmatic hernia in a single anesthetic procedure was feasible and curative in a dog. Subclinical diaphragmatic hernias diagnosed during surgery can be properly treated when adequate materials and trained professionals are available.

## Data Availability

The datasets generated during and/or analysed during the current study are available from the corresponding author on reasonable request.
